# Metagenomic next-generation sequencing reveals microbial community characteristics during acute exacerbations of interstitial pneumonia and their associations with clinical phenotypes

**DOI:** 10.3389/fcimb.2026.1809022

**Published:** 2026-05-28

**Authors:** Miao Ma, Linlin Wang, Min Chen, Shenyun Shi, Xianhua Gui, Xinmei Huang

**Affiliations:** 1 Department of Respiratory and Critical Care Medicine, Nanjing Drum Tower Hospital, Affiliated Hospital of Medical School, Nanjing University, Nanjing, China; 2 Dinfectome Inc., Nanjing, Jiangsu, China

**Keywords:** acute exacerbation, diagnosis, interstitial lung disease, lung microbiome, metagenomic next-generation sequencing

## Abstract

**Objective:**

Accurate pathogen detection is crucial for clinical management of interstitial lung diseases (ILDs), but conventional culture methods (CMT) have limited sensitivity. This study evaluated the diagnostic performance of metagenomic next-generation sequencing (mNGS) versus CMT in ILD patients and characterized differences in lower respiratory microbiome between stable (Stable) and acute exacerbation (AE) stage, as well as their associations with clinical indicators.

**Methods:**

We retrospectively analyzed ILD patients admitted between September 2021 and November 2023. Multidisciplinary discussion (MDT)-based comprehensive diagnosis served as the reference standard. We compared the sensitivity, specificity, and accuracy of mNGS and CMT. Microbiome analyses were performed to assess community composition and diversity in the Stable and AE groups, and to explore correlations with clinical features (e.g., frequency of exacerbations, oxygenation index, inflammatory markers).

**Results:**

The sensitivity of mNGS (95.60%) was significantly higher than that of CMT (32.20%). In 61.80% of patients, only mNGS yielded positive results, highlighting its diagnostic advantage. A total of 77 microorganisms were detected; bacteria accounted for 66.67% (e.g., Streptococcus pneumoniae, Haemophilus parainfluenzae). Among fungi, Candida albicans and Pneumocystis jirovecii predominated. Microbial diversity was significantly lower in the AE group than in the Stable group (*p* < 0.01). Candida albicans (*p* = 0.032) and Abiotrophia defectiva (p=0.011) were enriched in AE, whereas Haemophilus parainfluenzae (*p* = 0.038) and Prevotella pallens (*p* = 0.022) were more abundant in Stable. Correlation analyses showed that Candida albicans was positively associated with exacerbation frequency (*p* < 0.05), while Streptococcus salivarius correlated positively with the oxygenation index. Abiotrophia defectiva was positively associated with Erythrocyte Sedimentation Rate (ESR) and body temperature, but negatively associated with lymphocyte count.

**Conclusion:**

Patients in the AE group exhibited altered microbial community structures, and increased fungal colonization may be associated with disease progression, suggesting new targets for clinical intervention.

## Background

Interstitial lung disease (ILD) comprises a highly heterogeneous group of disorders characterized by inflammation and fibrosis of the pulmonary interstitium. More than 200 ILD entities have been identified, with etiologies involving environmental exposures, autoimmunity, and genetic susceptibility. Because clinical manifestations are nonspecific and imaging and pathological features are diverse, diagnosis and treatment require a multidisciplinary approach (Wijsenbeek et al., 2022) Acute exacerbations (AE-ILD) can occur at any stage of disease progression, and all ILD subtypes carry a potential risk of AE (Suzuki et al., 2020) Reported mortality after acute exacerbation of idiopathic pulmonary fibrosis is 80%–100%, the in-hospital mortality of connective tissue disease–associated ILD is 50%–100%, and that of hypersensitivity pneumonitis is 75%–100% (Salonen et al., 2020; Suzuki et al., 2020) The heterogeneity and complexity of ILD complicate clinical diagnosis and therapeutic decision-making. Accurate etiological diagnosis is crucial for guiding antimicrobial therapy, yet conventional microbiological testing (CMT) has low sensitivity, particularly for fastidious or non-culturable pathogens. Moreover, studies have shown that the composition of the microbiota is closely related to the onset and progression of pulmonary diseases (Li et al., 2024), but effective biomarkers and precision strategies are still lacking in ILD management.

In recent years, metagenomic next-generation sequencing (mNGS), an emerging high-throughput sequencing technology, has demonstrated significant value in the diagnosis of infectious diseases (Cretu et al., 2023) With its high sensitivity and specificity, mNGS can capture microbial information directly from clinical specimens, providing a more comprehensive characterization of the microbial community and offering new avenues for diagnosis and treatment (Szymanska et al., 2023; Lu et al., 2024)° However, the application of mNGS in ILD has not been fully explored. Current ILD diagnosis relies heavily on imaging and clinical presentation, which cannot comprehensively reflect the pulmonary microbial environment, and studies on microbiome changes across different disease stage of ILD and their correlations with clinical indicators remain scarce. Therefore, clarifying the dynamic changes of microbial communities across disease stage and their impact on clinical management is of great significance.

Based on bronchoalveolar lavage fluid (BALF) samples from 116 ILD patients, this study used mNGS to: (1) systematically compare the diagnostic performance of mNGS and conventional microbiological testing (CMT) in ILD-related pulmonary infections; (2) delineate microbiome differences between the stable and acute exacerbation stage; and (3) explore associations between the microbiome and clinical indicators to inform therapeutic strategies. These findings may reveal microbial dynamics across disease stage, provide a theoretical basis for individualized treatment, and advance precision medicine in ILD.

## Materials and methods

### Study participants and information collection

This retrospective analysis included 116 patients with ILD who were diagnosed and treated in the Department of Respiratory and Critical Care Medicine at Nanjing Drum Tower Hospital between September 2021 and November 2023 and underwent BALF-mNGS. Among them, 91 patients were in the stable (Stable) stage and 25 were in the acute exacerbation (AE) stage. Inclusion criteria were: (1) age ≥18 years; (2) diagnosis of ILD in accordance with international guidelines (e.g., ATS/ERS); and (3) concurrent BALF-mNGS and conventional microbiological testing (CMT) during the same hospitalization. Exclusion criteria were: (1) incomplete clinical data, including laboratory indices and high-resolution computed tomography (HRCT); (2) presence of malignancy; (3) follow-up duration <1 year; (4) availability of only non-BALF specimens (e.g., blood, sputum, pleural effusion, or tissue) without BALF; and (5) mNGS data failing predefined quality control and filtering criteria.

In this study, CMT was specifically defined as sputum culture and BALF culture, both performed in the hospital’s clinical microbiology laboratory according to standard procedures. PCR assays, special staining techniques, and serological tests were not included in the definition of CMT.

The final composite clinical diagnosis was established by two experienced clinicians based on clinical features together with mNGS and CMT results; discrepancies were adjudicated by a third expert. Data collected included age, sex, clinical symptoms, duration of fever, peak temperature, number of acute exacerbations, and outcomes. Laboratory results obtained prior to bronchoscopy were recorded, including white blood cell count, lymphocyte percentage (L%), neutrophil percentage (Neu%), C-reactive protein (CRP), erythrocyte sedimentation rate (ESR), procalcitonin (PCT), interleukin-6 (IL-6), lactate dehydrogenase (LDH), rheumatoid factor (RF), arterial partial pressure of oxygen (PaO2), arterial partial pressure of carbon dioxide (PaCO2), oxygen saturation (SpO2), and oxygenation index (PaO2/FiO2). The study protocol was approved by the Ethics Committee of Nanjing Drum Tower Hospital, affiliated with Nanjing University Medical School, on August 12, 2021 (approval No. 2024-JS-99).

### BALF collection

Bronchoscopy was performed under conscious intravenous sedation with midazolam. Topical anesthesia of the larynx, trachea, and carina was achieved with 2% lidocaine (Sanchine, China). The bronchoscope was wedged in the lesion’s segment or lobe, and the lavage was performed with three aliquots of sterile saline (Baxter, China), 1 mL/kg each, with a suction pressure of 100 mmHg. All BALF samples were then immediately processed and stored according to the requirements of the laboratory.

### DNA extraction and metagenomic sequencing

DNA was extracted using the TIANamp Magnetic DNA Kit (Tiangen) according to the manufacturer’s protocols. The quantity and quality of DNA were assessed using the Qubit (Thermo Fisher Scientific) and NanoDrop (Thermo Fisher Scientific), respectively. DNA libraries were prepared using the Hieff NGS C130P2 OnePot II DNA Library Prep Kit for MGI (Yeasen Biotechnology) according to the manufacturer’s protocols. Agilent 2100 was used for quality control and DNA libraries were 50bp single-end sequenced on DIFSEQ-200.

### Bioinformatics

An in-house bioinformatics pipeline was used for pathogen identification. Briefly, raw sequencing data underwent quality control by removing low-quality reads, adapter contamination, duplicated reads, and short reads (length <36 bp), generating high-quality sequencing data. Human host sequences were then identified and removed by mapping reads to the human reference genome (hs37d5) using Bowtie2 software (version 2.2.6). Reads that could not be mapped to the human genome were retained for downstream microbial analysis. The remaining non-human sequences were taxonomically classified using Kraken2 software against a custom microbial genome database (version 1.0.0). This comprehensive database incorporated bacterial, viral, fungal, and parasitic genomes sourced from GenBank (http://ftp.ncbi.nlm.nih.gov/genomes/genbank/).

Because Kraken2 is a k-mer–based taxonomic classification tool rather than a conventional sequence alignment–based method, no fixed sequence alignment similarity threshold was applied in the traditional sense. Species-level reporting was based on Kraken2 classification results combined with predefined read-count thresholds and background filtering criteria.

### Interpretation and reporting

The criteria for defining a positive detection were as follows: (1) at least one species-specific read was required for the detection of Mycobacterium, Nocardia, and Legionella pneumophila; (2) for other bacteria, fungi, viruses, and parasites, a minimum of three unique reads was required; and (3) pathogens were excluded if the ratio of reads per million (RPM) in the clinical sample to that in the no-template control (NTC) was <10. These thresholds were used to filter low-abundance taxa and reduce background contamination. Organisms meeting the above thresholds were subsequently interpreted for clinical relevance by integrating specimen type and clinical context.

Because this study focused on shotgun metagenomic sequencing for clinical pathogen detection rather than 16S rRNA or ITS amplicon sequencing, amplicon-oriented platforms such as QIIME2 and Mothur were not used for cross-validation.

### Statistical analysis

Statistical analyses were performed using SPSS software (IBM, version 25.0), and *P* < 0.05 was considered statistically significant. Additional analyses were conducted using R software (v4.0.1) with default parameters. Alpha diversity was estimated using the Shannon index based on the taxonomic profile of each sample. Beta diversity was assessed using the Bray-Curtis distance and compared between the Stable and AE groups using the Wilcoxon rank-sum test, followed by visualization with principal coordinate analysis (PCoA). PERMANOVA was performed using the R package “vegan” to assess differences in Bray-Curtis distances between the Stable and AE groups. Differential relative abundance of taxonomic groups at the genus level was tested using the Kruskal-Wallis rank-sum test (R package “kruskal.test”). For group comparisons, only species with a mean relative abundance >0.1% and penetrance >10% across all samples were included. Spearman’s correlations between clinical features and the relative abundances of genera were calculated using the R package “cor.test”, and all P values were adjusted using false discovery rate (FDR) correction. Statistically significant differences in microbial relative abundance among groups were further assessed using linear discriminant analysis effect size (LEfSe).

## Result

### Clinical characteristics

A total of 116 patients were included in this study, comprising a Stable group (n = 91) and an acute exacerbation (AE) group (n = 25). Demographic analyses showed no significant differences between the two groups in age (63.51 ± 11.10 years vs. 64.00 ± 12.87 years, *p* = 0.575), sex distribution (male proportion: 53.85% vs. 64.00%, *p* = 0.496), or BMI (22.52 ± 2.90 vs. 23.17 ± 1.91 kg/m², *p* = 0.140) (**Table 1**).

**Table 1:** Basic characteristics and clinic parameters of the Stable and AE group.

Characteristic	Stable group (n=91)	AE group (n=25)	*p*-value
Demographics			
Age (years)	63.51 ± 11.10	64.00 ± 12.87	0.575
Gender, n, (% male)	49 (53.85%)	16 (64.00%)	0.496
BMI (kg/m²)	22.52 ± 2.90	23.17 ± 1.91	0.140
Smoking, n, (%)	29 31.87%)	9(36.00%)	0.697
Disease classification			0.283
IPF, n (%)	19 (20.88%)	8 (32.00%)	
CTD-ILD, n (%)	40 (43.96%)	12 (48.00%)	
Other ILDs, n (%)	32 (35.17%)	5 (20.00%)	
Clinical parameters			
Duration of illness (days)	150 [517.50]	180 [640.00]	0.371
Peak temperature (°C)	37.67 ± 0.87	37.85 ± 0.84	0.197
Exacerbation frequency (times)	0.00 [1.00]	0.00 [1.00]	0.032
Treatment and exposure history			
Immunosuppressant, n, (%)	25 (27.47%)	5 (20.00%)	0.450
Prior antibiotic exposure, n, (%)	80 (87.91%)	25 (100.00)	0.118
Glucocorticoid, n, (%)	46 (50.55%)	15 (60.00%)	0.402
Laboratory results			
WBC (10^9/L)	7.46 ± 3.46	10.83 ± 5.64	0.002
Lymphocyte count (10^9/L)	1.49 ± 1.08	0.99 ± 0.87	0.001
Neutrophil count (10^9/L)	5.10 ± 2.86	9.39 ± 5.37	< 0.001
CRP (mg/L)	9.30 [34.85]	92.6 [115.70]	< 0.001
ESR (mm/h)	29.00 [47.00]	78.00 [55.00]	0.002
PCT (ng/mL)	0.04 [0.06]	0.16 [0.58]	< 0.001
LDH (U/L)	218.00 [83.00]	371.00 [338.00]	< 0.001
IL_6 (pg/mL)	28.35 [20.86]	28.35 [23.35]	0.969
RF (IU/mL)	0.00 [20.95]	0.00 [0.00]	0.236
PaO2 (mmHg)	83.57 ± 19.41	82.27 ± 23.07	0.223
PaCO2 (mmHg)	37.77 ± 4.88	37.28 ± 9.32	0.247
SpO2 (%)	95.89 ± 2.47	94.76 ± 3.50	0.050
PaO_2_/FiO_2_ (mmHg)	316.87 [93.03]	203.12 [85.05]	< 0.001

We further categorized the cohort by ILD etiology into idiopathic pulmonary fibrosis (IPF), connective tissue disease-associated ILD (CTD-ILD), and other ILDs. The distribution of etiological subtypes did not differ significantly between the Stable and AE groups (*p* = 0.283), with CTD-ILD representing the most common subtype in both groups (43.96% vs. 48.00%) (**Table 1**).

Potential clinical confounders that may affect the lower respiratory tract microbiome and the diagnostic performance of mNGS were also compared between groups. No significant between-group differences were observed in prior antibiotic exposure within 30 days (87.91% vs. 100.00%, *p* = 0.118), glucocorticoid use (50.55% vs. 60.00%, *p* = 0.402), immunosuppressant use (27.47% vs. 20.00%, p = 0.450), or smoking (31.87% vs. 36.00%, *p* = 0.697) (**Table 1**).

With respect to clinical parameters, the AE group had a significantly higher frequency of disease exacerbations (*p* = 0.032) and significantly elevated inflammatory markers, including neutrophil count (9.39 ± 5.37 vs. 5.10 ± 2.86 × 10^9/L, *p* < 0.001), CRP (92.60 [115.70] vs. 9.30 [34.85] mg/L, p < 0.001), ESR (78.00 [55.00] vs. 29.00 [47.00] mm/h, *p* = 0.002), PCT (0.16 [0.58] vs. 0.04 [0.06] ng/mL, *p* < 0.001), and LDH (371.00 [338.00] vs. 218.00 [83.00] U/L, *p* < 0.001). In contrast, the PaO_2_/FiO_2_ ratio was significantly lower in the AE group than in the Stable group (203.12 [85.05] vs. 316.87 [93.03] mmHg, *p* < 0.001), indicating impaired oxygenation (**Table 1**).

### Diagnostic performance of mNGS and CMTs in ILD patients

**Figure 1:**
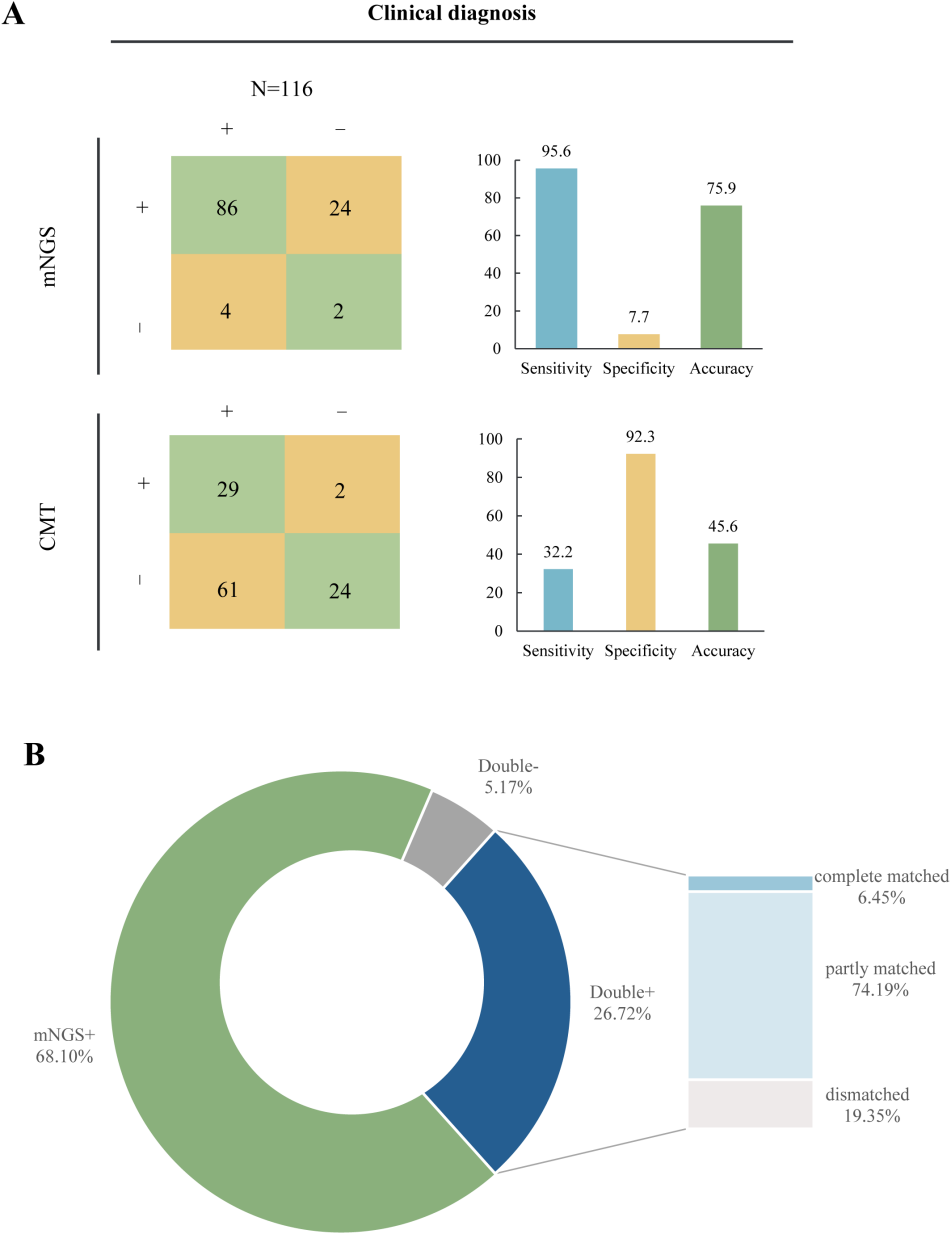
Comparison of results and consistency with cases of mNGS and CMT. **(A)** The 2×2 contingency tables comparing the performance of mNGS relative to CMT; **(B)** Comparison of Consistency between mNGS and CMT. *P < 0.05; **P < 0.01; ***P < 0.001.

Diagnostic performance was evaluated using the clinical composite diagnosis as the reference standard. Among 116 clinical samples, mNGS achieved a sensitivity of 95.60%, a specificity of 7.70%, and an accuracy of 75.90%. In contrast, CMT showed a sensitivity of only 32.20%, a specificity of 92.30%, and an accuracy of 45.60%, indicating that mNGS had substantially higher sensitivity and overall accuracy than CMT, whereas CMT showed markedly higher specificity (**Figure 1A**). In terms of species-detection concordance, 31 of 116 samples (26.72%) were positive by both mNGS and CMT, while 79 (68.10%) were positive only by mNGS and 6 (5.17%) were negative by both methods. Among the 31 double-positive samples, complete concordance was observed in 7 cases, partial concordance in 23 cases, and discordance in 6 cases. These results suggest that, although the two methods shared some overlap in pathogen detection, full agreement between mNGS and CMT remained limited ((**Figure 1B**).

**Figure 2:**
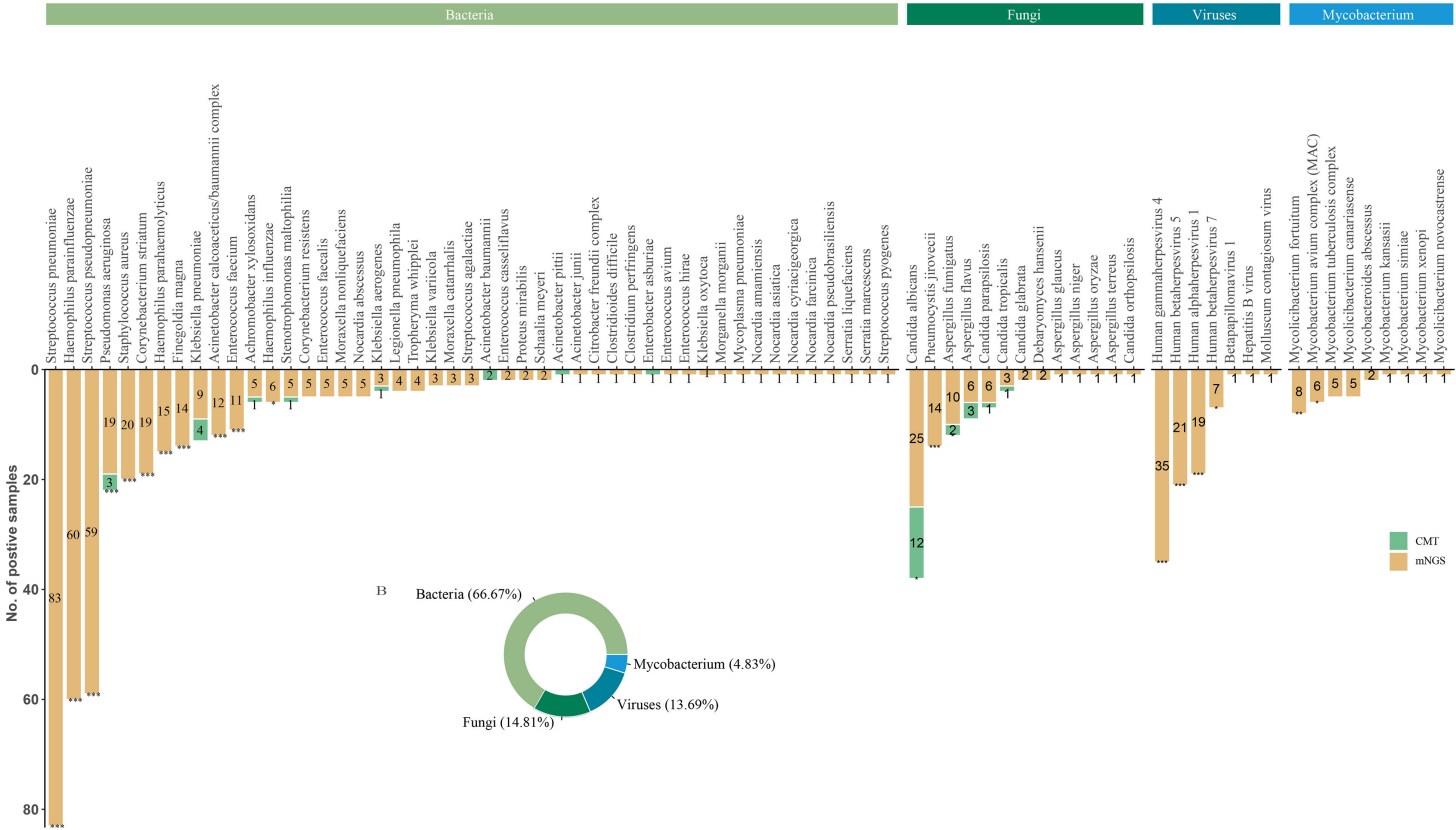
Pathogen identification based. The column chart showed all the organisms identified and reported.

A total of 77 distinct microbial species were identified by combining CMT and mNGS. Bacteria constituted the predominant group, accounting for 66.67% of all identified pathogens. The most prevalent bacteria, including Streptococcus pneumoniae, Haemophilus parainfluenzae, and Streptococcus pseudopneumoniae, were detected at significantly higher frequencies by mNGS than by CMT (*p* < 0.001). Significant differences (*p* < 0.05) were also observed for eight additional bacterial species, such as Streptococcus pneumoniae and Corynebacterium striatum, all of which were more frequently detected by mNGS. Among fungi pathogens, Candida albicans, Pneumocystis jirovecii, and Aspergillus fumigatus were the most common, with mNGS again demonstrating significantly higher detection rates (*p* < 0.001). Furthermore, seven viral species were detected exclusively by mNGS (**Figures 2A, B**).

### Microbiome analysis in the stable and AE groups

#### Community composition and differential comparisons

**Figure 3:**
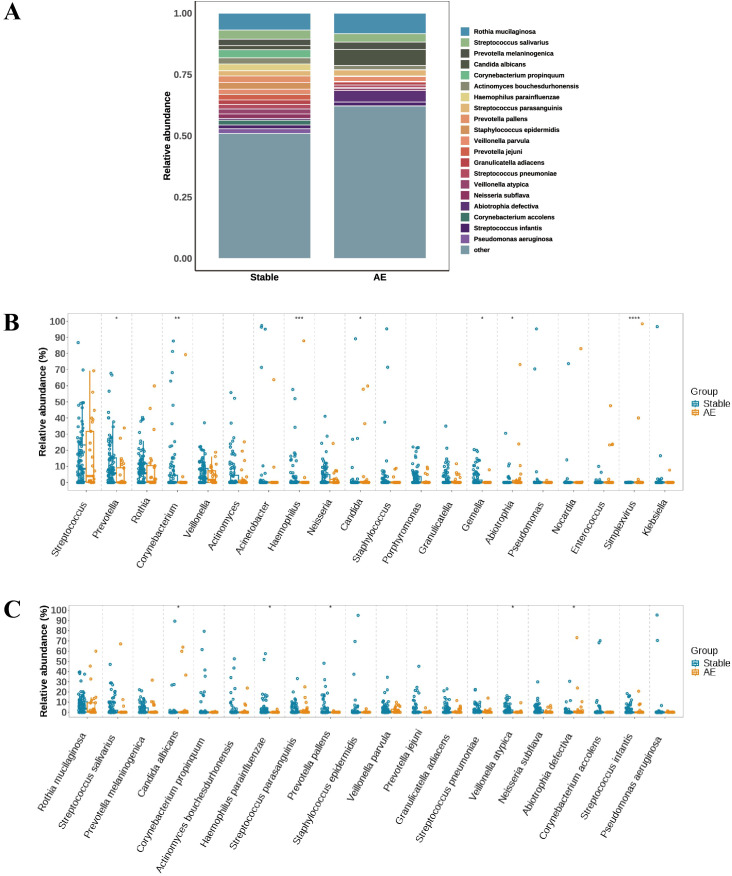
Comparison of microbial composition and relative abundance between the Stable and AE groups. **(A)** Relative abundance of major species in both groups at the species level. **(B)** Top 20 differentially abundant genera between groups. **(C)** Top 20 differentially abundant species between groups. *P < 0.05, **P < 0.01. *P < 0.05, **P < 0.01, and *P < 0.001.

Based on single-time-point BALF samples collected at the time of clinical evaluation, we compared the lower respiratory tract microbiome between the Stable and AE groups. At the species level, we compared taxonomic composition and relative abundances between groups. In the Stable group, *Rothia mucilaginosa* (6.86%) was most abundant, followed by *Streptococcus salivarius* (3.73%) and *Corynebacterium propinquum* (3.35%). Other predominant species included *Haemophilus parainfluenzae* (2.80%), Prevotella pallens (2.73%), *Staphylococcus epidermidis* (2.68%), and *Prevotella melaninogenica* (2.63%), indicating a community dominated by common mucosal commensals and opportunistic pathogens. In contrast, the AE group exhibited a different structure. Although *Rothia mucilaginosa* remained dominant, its relative abundance was higher (8.33%), followed by the fungus Candida albicans (6.58), which was not detected in the Stable group. Also relatively abundant were *Abiotrophia defectiva* (4.74%) and *Streptococcus salivarius* (3.43%), whereas *Prevotella melaninogenica* (2.99%) and *Veillonella parvula* (2.02%) were less abundant than in the Stable group ((**Figure 3A**).

We further compared the top 20 species by relative abundance between the Stable and AE groups. At the genus level, Candida (*p* = 0.030), Simplexvirus (*p* = 0.000), and Abiotrophia (*p* = 0.011) were significantly enriched in the AE group; Haemophilus (p = 0.000), Prevotella (*p* = 0.049), Gemella (*p* = 0.034), and Corynebacterium (*p* = 0.003) were significantly enriched in the Stable group (**Figure 3B**). At the species level, Abiotrophia defectiva (*p* = 0.011) and Candida albicans (*p* = 0.032) were significantly enriched in the AE group, whereas Haemophilus parainfluenzae (*p* = 0.038), Prevotella pallens (*p* = 0.022), and Veillonella parvula (*p* = 0.046) were significantly more abundant in the Stable group (**Figure 3C**). These findings indicate distinct ecological features in the AE group, characterized by increased fungal (C. albicans) abundance and altered bacterial community composition.

### Microbial diversity

**Figure 4:**
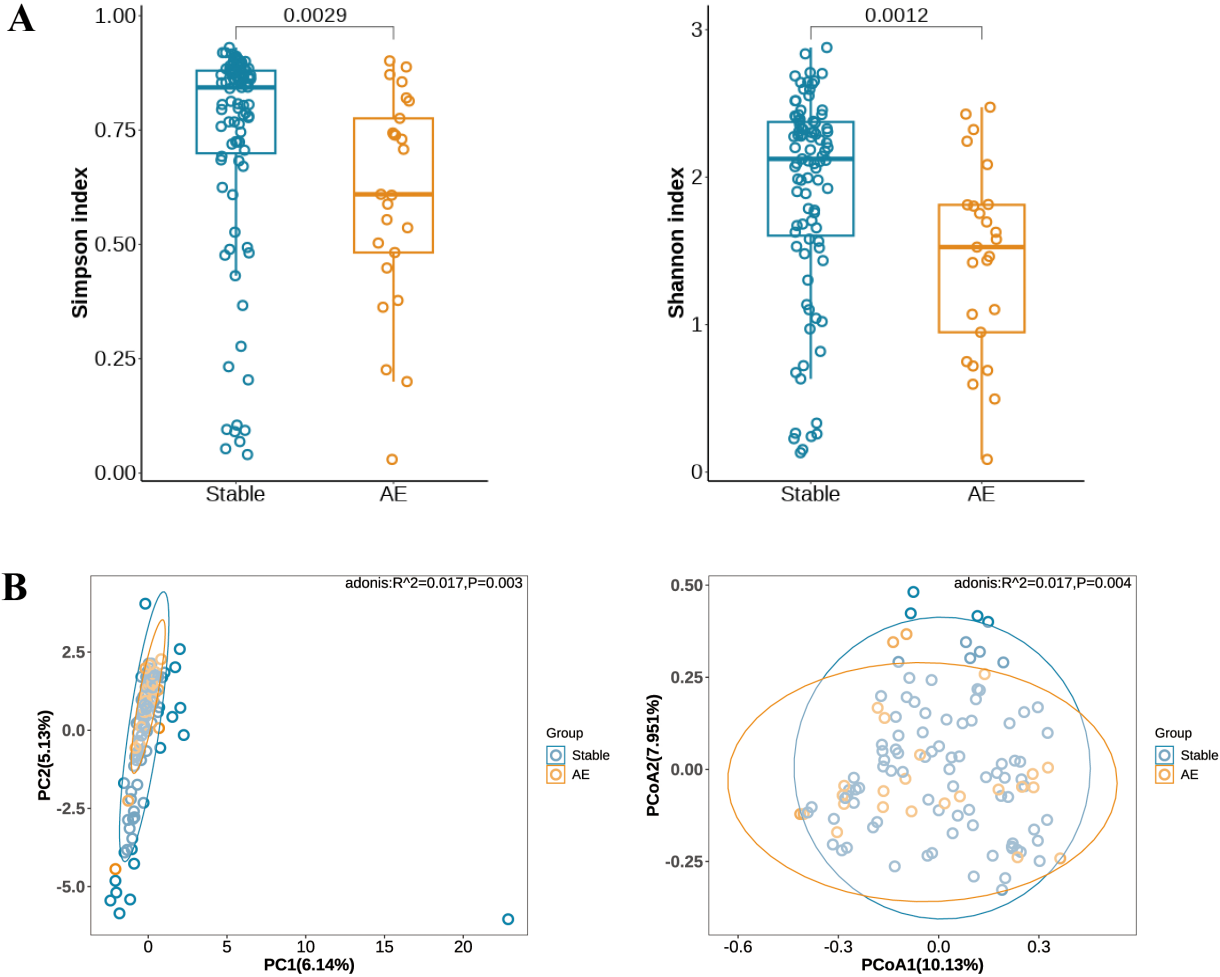
Alpha and beta diversity of the lower respiratory tract (LRT) microbiota in the Stable and AE groups. **(A)** Simpson index and Shannon index, both showing statistically significant differences between groups. Beta diversity estimated by **(B)** PCoA and PCA, also showed statistically significant differences between groups. PCoA, principal coordinate analysis; PCA, principal component analysis.

Alpha diversity based on the Shannon and Simpson indices was significantly lower in the AE group than in the Stable group (p < 0.01, Kruskal–Wallis test) (**Figure 4A**). In addition, PCA and PCoA analyses based on Bray–Curtis distances showed significant differences in beta diversity between the two groups (*p* < 0.01, ADONIS) (**Figure 4B**), indicating distinct lower respiratory tract community structures between patients sampled during the stable and acute exacerbation stages.

#### The dominant microbial taxa in the Stable and AE groups

**Figure 5:**
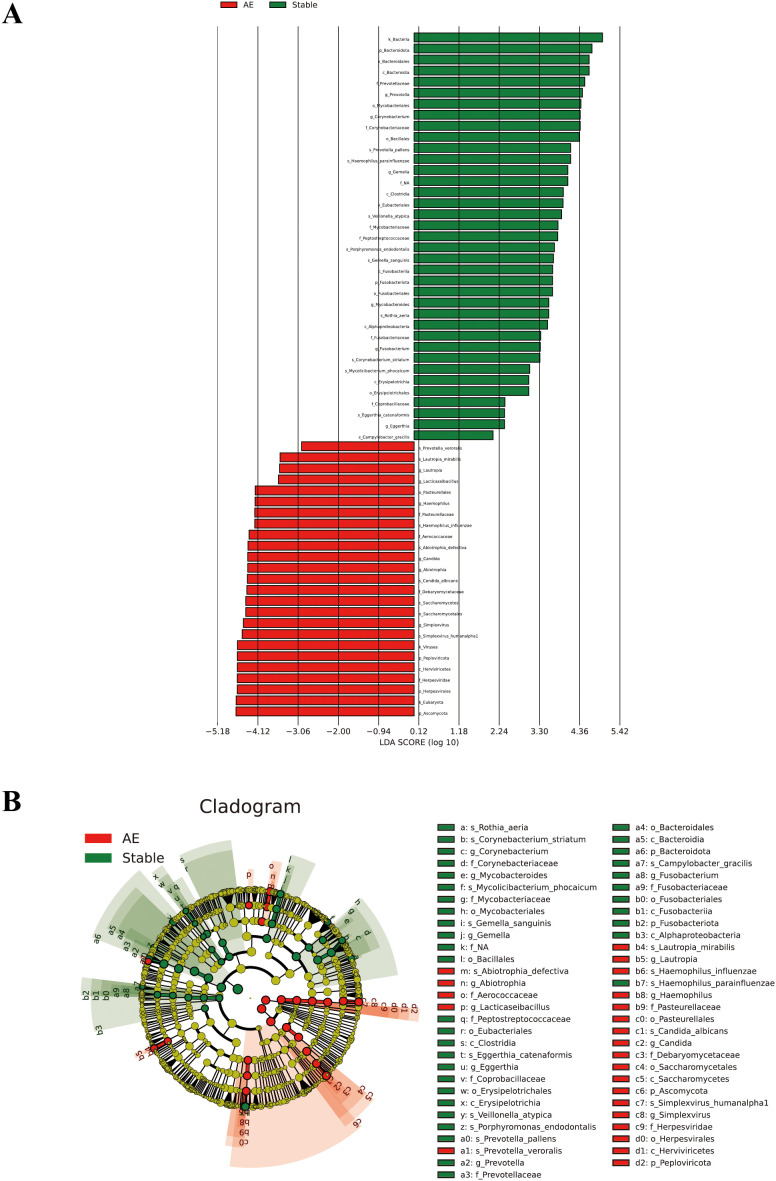
Linear discriminant analysis Effect Size (LEfSe). **(A)** LDA shows distinct lung microbiome composition associated with Stable and AE groups. LDA scores as calculated by LEfSe of taxa differentially abundant in different disease groups. Only taxa with LDA scores of more than two and p-value <0.05 are shown here. **(B)** Cladogram. Circles radiating from the inside out represent taxonomic levels from phylum to genus (or species). At different taxonomy levels, each small circle represents a classification belonging to that level, and the size of the small circle diameter is proportional to the size of the relative abundance. Coloring principle: no significant differences in species uniform coloring yellow Color, different species Biomarker follows group color, red nodes represent important microbial groups in the red group, green nodes represent important microbial groups in the green group.

Sixty-two discriminative features were identified using linear discriminant analysis effect size (LEfSe). Of these, 25 taxa were discriminative for AE patients and 37 taxa were discriminative for Stable patients (**Figures 5A, B**). Biomarker names, LAD scores and log values were shown in the figure.

At the genus level, Haemophilus was significantly enriched in both groups. The AE group was characterized by enrichment of Candida, Simplexvirus, and Defluvibacter, whereas the Stable group was dominated by Prevotella, Gemella, and Corynebacterium (LDA score [log10] > 4). At the species level, Haemophilus influenzae, Candida albicans, Human alphaherpesvirus 1, and Defluvibacter spp. were representative of the AE group, while Haemophilus parainfluenzae and Prevotella pallens predominated in the Stable group (LDA score [log10] > 4). These findings are consistent with the differential abundance analyses and further support distinct microbial community structures and potential pathogenic profiles between the AE and Stable groups (**Figures 5A, B**).

### Correlation between microbiota and clinical measures

**Figure 6:**
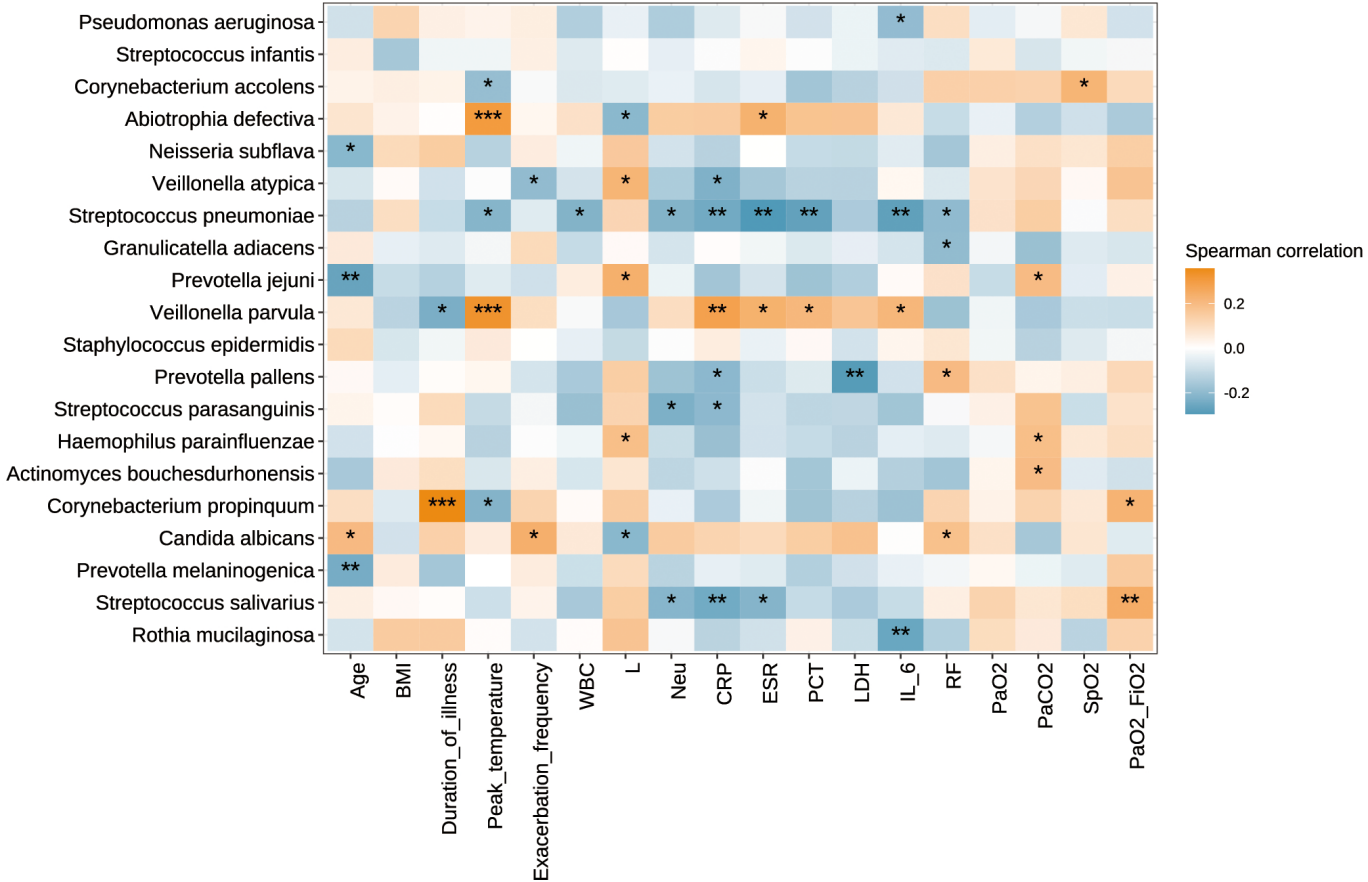
Heatmap of Spearman correlations between clinical measures and microbiota species. WBC (white blood cell count), NE (Neutrophil count), L (Lymphocyte count), ESR (Erythrocyte Sedimentation Rate), RF (Rheumatoid Factor), CRP (C-reactive protein), LDH (Lactate Dehydrogenase), PCT (procalcitonin), PaCO2 (Partial Pressure of Carbon Dioxide in Arterial Blood), PaO2/FiO2 (ratio of arterial PaO2 (mmHg) to fraction of inspired oxygen (FiO2)). *P < 0.05, **P < 0.01, ***P  <  0.001.

We assessed Spearman correlations between the relative abundances of the top 20 most prevalent species and clinical features (demographics, laboratory tests, and arterial blood gas parameters). Abiotrophia defectiva showed positive correlations with peak temperature and ESR (*p* < 0.05) and a negative correlation with lymphocyte percentage (L) (*p* < 0.05). Candida albicans correlated positively with age, exacerbation frequency, and rheumatoid factor (RF) (*p* < 0.05), and negatively with L (p < 0.05). Haemophilus parainfluenzae correlated positively with L and PaCO2 (*p* < 0.05). Prevotella pallens correlated positively with RF (*p* < 0.05) and negatively with C-reactive protein (CRP) and lactate dehydrogenase (LDH) (*p* < 0.05). In addition, Veillonella atypica was negatively associated with exacerbation frequency, while Streptococcus salivarius and Corynebacterium propinquum were both positively associated with the oxygenation index (PaO2/FiO2) (**Figure 6**).

## Discussion

In recent years, breakthroughs in microbial identification technologies have not only greatly improved the sensitivity of pathogen detection but also broadened our understanding of the abundance and diversity of the human microbiome, offering new perspectives for investigating the roles of microbial communities in pulmonary health and disease (Dickson et al., 2013; Moffatt and Cookson, 2017). In the highly complex and heterogeneous field of ILD, conventional microbiological methods (e.g., culture) often fail to clarify etiologies due to limited sensitivity. The application of mNGS has effectively overcome this bottleneck. Through an unbiased detection strategy, mNGS simultaneously covers both culturable and fastidious organisms, markedly increasing the detection rate and accuracy of pathogen identification (Zheng et al., 2021). Notably, accumulating evidence indicates that respiratory microbial dysbiosis plays an important role in the pathogenesis of ILD (He et al., 2022; Zhang et al., 2024). On this basis, a systematic assessment of the clinical value of mNGS in ILD diagnosis and management is of considerable significance for advancing precision medicine in ILD. In this study, we innovatively applied mNGS to conduct systematic pathogen identification and microbiome profiling of respiratory samples from ILD patients during stable and acute exacerbation stage. We focused on comprehensively evaluating the clinical utility of mNGS in ILD diagnosis and exploring the relationships between microbiome features across disease stage and clinical indicators, with the aim of providing a theoretical and practical foundation for early diagnosis and individualized treatment.

In our cohort, the sensitivity of mNGS for ILD-associated infections was markedly higher than that of conventional culture (95.6% vs. 32.2%), whereas its specificity was substantially lower (7.7% vs. 92.3%). This discrepancy should be interpreted in light of how mNGS positivity was defined in the present study. Specifically, an mNGS result was considered positive when the final laboratory report identified at least one reportable microorganism rather than when the detected organism was clinically adjudicated as the definite etiologic pathogen of the current infectious episode. Therefore, the calculated specificity reflects the agreement between laboratory-level reportable microorganism detection and the final composite clinical diagnosis, rather than the specificity of clinically confirmed pathogen identification. In BALF specimens, the high analytical sensitivity of mNGS allows detection not only of true pathogens but also of oral commensals, airway colonizers, opportunistic organisms, and residual microbial nucleic acids from previous or non-active infections. As a result, when the final clinical assessment did not support active infection, such cases were statistically categorized as false positives, thereby lowering the measured specificity of mNGS. This issue may be particularly relevant in ILD, where structural lung abnormalities, chronic inflammatory backgrounds, and airway microbiome dysbiosis may further increase the likelihood of detecting microorganisms not directly responsible for the current clinical syndrome (Chiu and Miller, 2019; Gu et al., 2019; Xu et al., 2026). Thus, the low specificity observed in our study more likely reflects the discrepancy between reportable microorganism detection and clinical confirmation of active infection, rather than simply indicating a high rate of environmental contamination or complete failure of the noise-reduction algorithm.

By contrast, CMT is largely culture-based and generally requires viable organisms at sufficient burden, resulting in a higher positivity threshold and a more conservative positivity profile. This may partly explain its higher specificity but lower sensitivity. Taken together, mNGS should be regarded as a highly sensitive adjunctive diagnostic tool rather than a standalone indicator of active infection, and its results must be interpreted in conjunction with clinical presentation, imaging findings, inflammatory markers, and conventional microbiological testing. Importantly, mNGS still offers substantial clinical advantages. Owing to its unbiased amplification and broad-spectrum detection, it can rapidly identify pathogens that are difficult to culture or target by conventional methods, including rare bacteria, fungi, and viruses (Zheng et al., 2021). Moreover, for most mNGS platforms, the average turnaround time from sample receipt to report is approximately 24 hours (Han et al., 2023), considerably shorter than the 48–72 hours typical of culture, thereby providing important support for early diagnosis and timely clinical decision-making.

We found that bacteria remained the dominant etiologic agents of pneumonia, with Streptococcus pneumoniae and Haemophilus parainfluenzae most frequently detected. These species can conditionally colonize the respiratory tract or nasopharynx (Hartzell et al., 2003) and pose risks to young children and older adults with comorbidities (Garriss et al., 2019). Candida albicans was the most frequently detected fungus (Li et al., 2019) and is often associated with invasive pulmonary infection and polymicrobial disease (Zhao et al., 2021). In addition, nine viruses detected by mNGS (e.g., human herpesvirus 1) suggest a potential role for viral–bacterial/fungal coinfections in pneumonia; the clinical manifestations of these viral infections often vary with age and immune status (Griffiths et al., 2015; Nowalk and Green, 2016), and causal mechanisms warrant further investigation.

Respiratory microbes are thought to contribute to respiratory physiology and the maturation and maintenance of the immune system, thereby helping to preserve homeostasis in the respiratory tract (Man et al., 2017). Shifts in microbial community structure reflect the pathophysiological processes of disease progression. Accordingly, we analyzed the lung microbiome using BALF-mNGS data, including taxonomic composition, alpha diversity, and correlations between high-prevalence species and clinical indicators. The Stable group was characterized by commensals and opportunistic pathogens (e.g., Haemophilus parainfluenzae, Prevotella pallens), which may help maintain respiratory microecological balance. In contrast, the AE group showed marked structural alterations (e.g., increased C. albicans abundance to 6.58% and decreased Prevotella), potentially reflecting links between fungal enrichment and immune dysregulation, as well as bacterial community reorganization and inflammation-driven mechanisms (Mirmonsef et al., 2012; Richardson and Moyes, 2015; Larsen, 2017).

Alpha diversity analyses showed significantly lower Shannon and Simpson indices in the AE group than in the Stable group, suggesting that reduced diversity may reflect intensified host immune responses during acute exacerbations or suppression of commensal communities by antibiotic therapy (Segal et al., 2016). Studies have further indicated that decreased respiratory microbial diversity may be associated with heightened pulmonary inflammation and may promote disease progression by impairing immunoregulatory functions (Mikhail and O’Dwyer, 2025). Beta diversity analyses (PCA and PCoA based on Bray–Curtis distances) confirmed significant structural differences between groups (p < 0.01), indicating substantial alterations in airway community composition during acute exacerbation. Similar phenomena have been reported in patients with acute exacerbations of idiopathic pulmonary fibrosis (IPF); for example, Han et al. (2021) observed increased abundances of potentially pathogenic taxa (e.g., Streptococcus and Haemophilus) and decreased commensals (e.g., Prevotella) during IPF exacerbations (Han et al., 2014). Such dysbiosis may exacerbate lung injury by amplifying local inflammatory responses or disrupting mucosal barrier function (Fastrès et al., 2017).

Moreover, the abundance of Candida albicans was significantly positively correlated with exacerbation frequency (p < 0.05), and its positive correlations with age and rheumatoid factor (RF) further suggest its potential as a predictive marker of AE in ILD. Prior studies indicate that fungal colonization may aggravate alveolar epithelial damage and fibrosis by triggering excessive release of proinflammatory mediators (e.g., IL-17, TNF-α) (Invernizzi et al., 2020; Lopes and Lionakis, 2022). Enrichment of Abiotrophia defectiva was positively correlated with peak temperature and ESR but negatively correlated with leukocyte counts, a seemingly paradoxical pattern that may reflect dual actions: promoting local inflammation via TLR signaling while potentially inducing “immune paralysis” by impairing neutrophil function, thereby increasing infection risk (Koo et al., 2017; Di Pierro et al., 2022). These findings suggest that overgrowth of opportunistic pathogens may jointly drive AE-ILD through proinflammatory and immunosuppressive mechanisms (Enaud et al., 2020).

Conversely, putatively beneficial commensals were associated with better oxygenation or lower inflammatory tone. Streptococcus salivarius and Corynebacterium propinquum correlated positively with the oxygenation index (PaO2/FiO2), suggesting potential roles in maintaining the alveolar–capillary barrier and gas exchange via metabolic products (e.g., short-chain fatty acids, hydrogen sulfide) and mucosal barrier/antioxidant pathways (Wallace et al., 2012; MacDonald et al., 2021; Drigot and Clark, 2024)°Prevotella pallens correlated negatively with CRP and LDH but positively with RF, implying a complex interface between systemic autoimmunity and local inflammation; prior literature shows Prevotella can produce butyrate and modulate NF−κB signaling and Treg/Th17 balance to alleviate inflammation (Larsen, 2017; Dickson, 2018; Liu et al., 2024).

Our data indicate a proinflammatory–anti-inflammatory imbalance in the respiratory microbiota during AE-ILD, characterized by enrichment of opportunistic pathogens (e.g., C. albicans and A. defectiva) and depletion of commensals (e.g., S. salivarius and Prevotella). This pattern highlights potential biomarkers and therapeutic targets for precision intervention.

This study has several limitations. First, the relatively small sample size and single-center retrospective design may limit the generalizability of our findings. Second, although no significant between-group differences were observed in prior antibiotic exposure, glucocorticoid use, immunosuppressant use, or smoking, residual confounding cannot be excluded because stratified analyses and multivariable-adjusted microbiome analyses were not performed; in addition, prior pulmonary infection history was incompletely documented. Third, although the cohort was categorized into IPF, CTD-ILD, and other ILDs, etiology-based subgroup microbiome analyses were not performed because of limited subgroup sample sizes. Finally, because this was a cross-sectional study based on single-time-point BALF sampling and without a healthy control cohort, the observed microbiome differences should be interpreted as cross-sectional associations rather than temporal or causal relationships, and ILD-specific dysbiosis could not be definitively distinguished from the core healthy lung microbiome. Future multicenter prospective studies with larger, better-characterized cohorts, serial sampling, and appropriate healthy controls are needed to validate these findings and clarify microbiome patterns across specific ILD subtypes.

## Conclusions

Our data indicate mNGS outperformed conventional microbiological testing by yielding higher sensitivity and a broader pathogen spectrum across bacteria, fungi, and viruses. Microbiome profiling revealed significant dysbiosis during AE, with reduced alpha diversity and clear beta-diversity separation. AE was enriched for Candida, Simplexvirus, and opportunists (e.g., Abiotrophia, Defluvibacter), whereas the Stable stage was dominated by commensals (Prevotella, Gemella, Corynebacterium). Key taxa correlated with clinical severity, linking AE-associated shifts to inflammatory load and oxygenation impairment. Integrating mNGS with microbiome signatures may facilitate identification of infection-related exacerbations and inform personalized management.

## Data Availability

The datasets presented in this study can be found in online repositories. The raw sequence data of mNGS in this study was deposited in the Genome Sequence Archive under accession number PRJCA045247.
